# Distinguishing the Associations Between Evening Screen Time and Sleep Quality Among Different Age Groups: A Population-Based Cross-Sectional Study

**DOI:** 10.3389/fpsyt.2022.865688

**Published:** 2022-06-24

**Authors:** Long Sun, Keqing Li, Lili Zhang, Yunshu Zhang

**Affiliations:** ^1^Centre for Health Management and Policy Research, School of Public Health, Cheeloo College of Medicine, Shandong University, Jinan, China; ^2^Key Laboratory of Health Economics and Policy Research, National Health Commission of China, Shandong University, Jinan, China; ^3^Hebei Provincial Mental Health Center, Baoding, China

**Keywords:** evening screen time, sleep quality, age differences, population-based study, China

## Abstract

**Objective:**

The age differences in the association between screen time and sleep problems have been implied in many studies, and this study aims to distinguish the associations between evening screen time and sleep quality among different age groups.

**Methods:**

This is a population-based, cross-sectional study among community residents aged ≥18 years in China. A total of 21,376 valid questionnaires were analyzed. Sleep quality was measured by the Pittsburgh Sleep Quality Index. Averaged evening screen time (AEST), sociodemographic information, and health-related behaviors were also evaluated in this study.

**Results:**

In the 18-to-34-year age group, compared with people without AEST, ≤1 h/day (β = 0.34, *p* < 0.05) and >3 h/day (β = 1.05, *p* < 0.001) of AEST were significantly associated with poor sleep quality, and a reverse S-shaped relationship for this association was shown. In the 35-to-49-year and 50-to-64-year age groups, ≤1 h/day (β = 0.43 and 0.36, both *p* < 0.001), ≤2 h/day (β = 0.43 and 0.31, *p* < 0.001 and *p* < 0.01), ≤3 h/day (β = 0.62 and 0.61, both *p* < 0.001), and >3 h/day (β = 1.55 and 1.88, both *p* < 0.001) of AEST were positively associated with poor sleep quality. In the 65-year-and-older age group, a J-shaped relationship was found, and ≤3 h/day (β = 0.82, *p* < 0.001) and >3 h/day (β = 1.84, *p* < 0.001) of AEST were associated with poor sleep quality.

**Conclusion:**

Associations between AEST and sleep quality among different age groups are different. In the 18-to-34-year and 65-year-and-older age groups, acceptable AEST is not related to sleep quality. In the 35-to-49-year and 50-to-64-year age groups, AEST was harmful to sleep quality.

## Introduction

Nowadays, screen-based electronic products have been an integral part of our lives. Globally, it was estimated that 4.57 billion people use the Internet until the year 2020, and this number is increasing rapidly (1). Although we must acknowledge that these screen-based electronic products bring convenience and pace to our lives, several negative health outcomes, such as obesity (2–4), poor quality of life (5–7), and sleep problems (8–10), also accompany these products (11).

Until now, sleep problems, one of the negative health outcomes of screen-based electronic product use, have been explored in many studies (12–14). However, a majority of these studies were conducted on children and adolescents (15–17), and the strong and consistent association between screen-based electronic product use and widespread sleep problems has also been identified among them (18). Recently, there have been a few studies targeting other age populations. Among elders, a study in Shaanxi Province, China, showed a negative relationship between using electronic devices before sleep and sleep quality (19). Several studies have focused on a wide age range of sleep problems. Two studies conducted among adults in the United States supported that more screen time was related to trouble falling asleep and waking during the night (20, 21). Another study conducted in Chinese Macao revealed a “J”-shaped relationship between sleep quality and the duration of television viewing, computer usage, and mobile phone usage (22). All of these studies supported that the high level of screen time was associated with poor sleep quality among elders and a wide range of age groups. However, the age differences in the associations between screen-based electronic product use and sleep problems were neglected in all of these studies.

As we know, both screen time and sleep problems were significantly different among different age groups. Regarding sleep problems, many studies have provided evidence that sleep problems increased with age (23–25). However, the daily screen time was decreased with age, which was also supported in a previous study (26). The different associations between sleep problems and screen time with age remind us that analyzing the association between sleep quality and screen time in a wide range of age groups may cause some bias in the results, and analyzing the associations between screen time and sleep problems among different age groups may be helpful for us to further understand the associations. But, to the best of our knowledge, the differences in the associations among different age groups have not yet been reported.

In contrast, evening screen time can also be seen as a relaxing method for many people, and an appropriate screen time may not be harmful to health, which was also recommended in previous studies (27, 28). A study of Canadian residents found that an increase in screen time did not occur at the expense of sleep duration between 1998 and 2010 (26). A recent systematic review also recommended that the screen time for adolescents at 2 h/day or less was not related to sleep problems (29), and a J-shaped relationship between sleep quality and the duration of screen time was also supported in a previous study in a wide range of age groups (22). Based on these previous findings, dividing screen time into different levels is also necessary to understand the real associations between screen time and sleep problems among different age groups, which is also not yet reported.

To fill the gaps, a population-based, cross-sectional study was conducted to distinguish the association between different levels of evening screen time and sleep quality in different age groups. It was helpful for us not only to further understand the association between screen time and sleep quality among different age groups but also to make a recommendation for the appropriate screen time to avoid poor sleep quality among different age groups.

## Methods

### Study Sample and Design

This is a cross-sectional study conducted among community residents aged 18 years and above in Chinese Hebei Province from June to August 2018, and a three-stage stratified cluster sampling was used to analyze the participants according to the following steps. First, simple random sampling was used to select the cities from all the 11 cities in Hebei Province, and five cities (i.e., Shijiazhuang, Baoding, Xingtai, Zhangjiakou, and Qinhuangdao) were selected in this step. Second, simple random sampling was repeatedly used to select the counties (rural region) and districts (urban region). In each selected city, three counties and one district were selected in this step. Third, one township or sub-district was randomly selected from each selected county or district. Fourth, one village or community was randomly selected in each selected township or sub-district. In total, 15 rural villages and 5 urban communities were selected. In the selected villages and communities, all the residents aged 18 and above were asked to participate in this study, and 21,376 valid questionnaires were collected.

### Interviewing Procedures

Before the survey, all the interviewers were trained for 2 days to make sure they had fully understood the research and questionnaire. The subjects were first visited by the village (community) administration. After the participants’ agreement with a written informed consent form, a face-to-face interview was scheduled by one interviewer. After completing the interview for each participant, the interviewer was asked to check the questionnaire, and there were two supervisors to check all the questionnaires to ensure the quality of these questionnaires. If there were missing data or logical problems in the questionnaires, the interviewers were asked to revisit or call the participants the next day. The study protocol was approved by the Institutional Review Board (IRB) of ^∗∗∗^ before data collection (Ref. No. 201813). Written informed consent was obtained from all the participants.

### Measures

#### Sleep Quality

Sleep quality was measured by the Chinese version of the Pittsburgh Sleep Quality Index (PSQI). It has been proven to be a tool with good reliability and validity to measure sleep quality in many previous studies worldwide (30–32). In this scale, there were 19 items to evaluate sleep quality, sleep latency, sleep duration, sleep efficiency, sleep disturbance, use of sleep medication, and daytime function of the subjects. The sum of these seven component scores was used to measure the subjects’ sleep quality. The higher values of sum scores presented poorer sleep quality (33, 34). In this study, the total scores were analyzed as a continuous variable.

#### Averaged Evening Screen Time

Averaged evening screen time (AEST) was measured by the following questions. First, all the participants were asked whether they used electronic products in their evening time. The answer could be chosen from yes or no. For the participants with a positive answer (yes), we further asked them what kinds of electronic products they used. After this, they were also asked about the average time per day they used these electronic products. In this study, we only analyzed the electronic products with screens. Participants only using electronic products without screens were recoded into no evening screen time. AEST was coded into no evening screen time (0), ≤1 h/day (1), ≤2 h/day (2), ≤3 h/day (3), and >3 h/day (4).

#### Health-Related Behaviors

In this study, health-related behaviors comprised smoking, drinking, and physical activity. Smoking was evaluated by the question that if they smoked. Participants who smoked recently or for more than 6 months in the previous years were coded as smoking (1). Drinking was evaluated by the question that if they drank. Participants who drank more than one time per week were coded as drinking (1). Physical activity was evaluated by the frequency of physical activity, and the answers were ≥3 times/week (1), 1–2 times/week (2), 1–3 times/month (3), and < 1 time/month (4).

#### Sociodemographic Variables

Age was calculated by the date of birth of the participants. This study aimed to analyze the different associations between AEST and sleep quality, and the participants were categorized into the following age groups: 18-to-34-year age group (1), 35-to-49-year age group (2), 50-to-64-year age group (3), and 65-year-and-older age group (4). Gender was assessed by male (1) and female (0). Ethnicity was assessed by Hans (1) and others (0). Education level was assessed by the question about the participants’ academic degrees. The answers were illiteracy, elementary school, middle school, high school, junior college, bachelor, and above. Due to the small percentage of the last three answers, education was recoded into illiteracy (1), elementary school (2), middle school (3), and high school or above (4). Married status was evaluated through one question about the participants’ married status with the answers never married, married, divorced, widowed, deuterogamist, and others. Due to the small percentage of the last four answers, married status was recoded into unmarried (1), married (2), and others (3). Region was assessed by asking the region where the participants lived, and the answers were rural (1) and urban (0).

### Statistical Methods

Data analysis was conducted by SPSS (IBM, Chicago, IL, United States) for Windows 24.0 (web version). A one-way analysis of variance and a chi-square test were conducted to compare the means or proportions among different age groups. Multiple linear regression analyses were performed to examine the association between AEST and sleep quality in different age groups. Charts were drawn by SPSS (IBM, Chicago, IL, United States), and spline interpolation was used to fill the line among different groups of AEST. All significance tests were two-tailed, and a *p*-value of 0.05 or less was considered statistically significant.

## Results

In this population-based, cross-sectional study, 21,376 valid participants were analyzed. To differentiate the associations between AEST and sleep quality in different age groups, the whole sample was divided into four groups, namely, 18-to-34-year age group (4,424/21,376, 20.7%), 35-to-49-year age group (4,970/21,376, 23.2%), 50-to-64-year age group (7,282/21,376, 34.1%), and 65-year-and-older age group (4,700/21,376, 22.0%). Table 1 summarizes that both sleep quality (*F* = 700.02, *p* < 0.001) and AEST (χ^2^ = 1,565.02, *p* < 0.001) were lower in higher age groups. Among these four age groups, gender (χ^2^ = 25.58, *p* < 0.001), education (χ^2^ = 4,872.71, *p* < 0.001), married status (χ^2^ = 5,156.31, *p* < 0.001), region (χ^2^ = 48.29, *p* < 0.001), smoking (χ^2^ = 114.32, *p* < 0.001), drinking (χ^2^ = 170.09, *p* < 0.001), and physical activity (χ^2^ = 452.90, *p* < 0.001) were statistically significant. The detailed information is summarized in Table 1.

**TABLE 1:** The socio-demographic, lifestyle, sleep quality, and averaged evening screen time (AEST) characteristics of the sample in different age groups.

Variables	Total [Mean ± SD/n (%)]	18–34 years old [Mean ± SD/n (%)]	35–49 years old [Mean ± SD/n (%)]	50–64 years old [Mean ± SD/n (%)]	≥65 years old [Mean ± SD/n (%)]	*F/*χ^2^
Observations	21,376 (100.0)	4,424 (20.7)	4,970 (23.3)	7,282 (34.1)	4,700 (22.0)	–
**Gender**						25.58***
Male	9,839 (46.0)	2,140 (48.4)	2,360 (47.5)	3,273 (44.9)	2,066 (44.0)	
Female	11,537 (54.0)	2,284 (51.6)	2,610 (52.5)	4,009 (55.1)	2,634 (56.1)	
**Ethnicity**						5.56
Hans	20,094 (94.0)	4,136 (93.5)	4,697 (94.5)	6,858 (94.2)	4,403 (93.7)	
Others	1,282 (6.0)	288 (6.5)	273 (5.5)	424 (5.8)	297 (6.3)	
**Education**						4872.71***
Illiteracy	2,691 (12.6)	55 (1.2)	187 (3.8)	1,014 (13.9)	1,435 (30.5)	
Elementary	5,264 (24.6)	371 (8.4)	1,123 (22.6)	1,951 (26.8)	1,819 (38.7)	
Middle school	8,274 (38.7)	1,921 (43.4)	2,571 (51.7)	2,778 (38.1)	1,004 (21.4)	
High school/above	5,147 (24.1)	2,077 (46.9)	1,089 (21.9)	1,539 (21.1)	442 (9.4)	
**Married Status**						5156.31***
Unmarried	1,548 (7.2)	1,228 (27.8)	127 (2.6)	134 (1.8)	59 (1.3)	
Married	18,487 (86.5)	3,144 (71.1)	4,761 (95.8)	6,845 (94.0)	3,737 (79.5)	
Others	1,341 (6.3)	52 (1.2)	82 (1.6)	303 (4.2)	904 (19.2)	
**Region**						48.29***
Urban	5,100 (23.9)	921 (20.8)	1122 (22.6)	1817 (25.0)	1240 (26.4)	
Rural	16,276 (76.1)	3503 (79.2)	3848 (77.4)	5465 (75.0)	3460 (73.6)	
**Smoking**						114.32***
Yes	4822 (22.6)	1029 (23.3)	1221 (24.6)	1779 (24.4)	793 (16.9)	
No	16554 (77.4)	3395 (76.7)	3749 (75.4)	5503 (75.6)	3907 (83.1)	
**Drinking**						170.09***
Yes	4956 (23.2)	1065 (24.1)	1392 (28.0)	1703 (23.4)	796 (16.9)	
No	16420 (76.8)	3359 (75.9)	3578 (72.0)	5579 (76.6)	3904 (83.1)	
**Physical activity**						452.90***
<3 times/week	9090 (42.5)	2394 (54.1)	2313 (46.5)	2715 (37.3)	1668 (35.5)	
≥3 times/week	12286 (57.5)	2030 (45.9)	2657 (53.5)	4567 (62.7)	3032 (64.5)	
**Sleep quality**	4.60 ± 3.54	3.01 ± 2.49	3.88 ± 2.94	5.27 ± 3.69	5.81 ± 3.97	700.02***
**AEST (hours/day)**						1565.02***
0	4710 (22.0)	416 (9.4)	809 (16.3)	1920 (26.4)	1565 (33.3)	
≤1	7077 (33.1)	1196 (27.0)	1781 (35.8)	2632 (36.1)	1468 (31.2)	
≤2	6756 (31.6)	1729 (39.1)	1741 (35.0)	2037 (28.0)	1249 (26.6)	
≤3	2207 (10.3)	821 (18.6)	539 (10.8)	531 (7.3)	316 (6.7)	
>3	626 (2.9)	262 (5.9)	100 (2.0)	162 (2.2)	102 (2.2)	

Averaged evening screen time denotes average evening screen time. ***p < 0.001.

**FIGURE 1:**
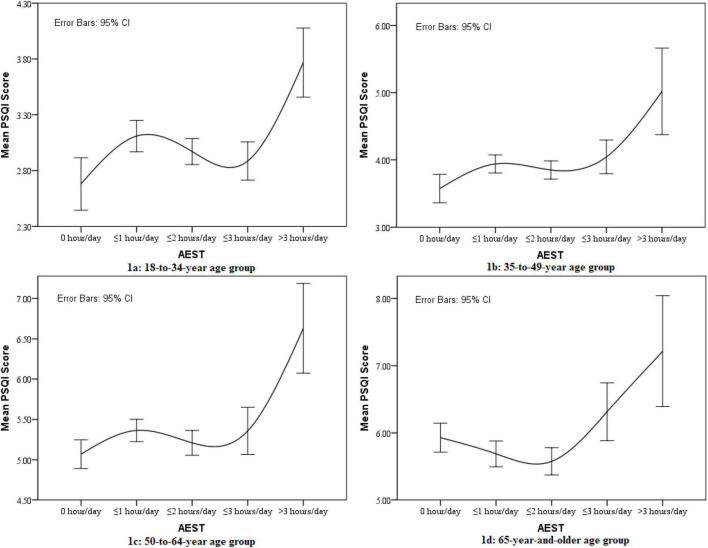
Line chart for the association between averaged evening screen time (AEST) and sleep quality in different age groups.

Figure 1 depicts the association between AEST and poor sleep quality by line charts in each age group. In this figure, a reverse S-shaped relationship between AEST and poor sleep quality was shown for the 18-to-34-year age group (a). In the 35-to-49-year age group (b) and 50-to-64-year age group (c), a consistent positive relationship between AEST and poor sleep quality was shown, whereas the relationship between AEST and poor sleep quality showed a J-shaped relationship for the 65-year-and-older age group (d).

Multiple linear regression analyses were conducted to explore the associations between AEST and poor sleep quality in the whole sample and each age group, as summarized in Table 2. In the whole sample, all classifications of AEST (β = 0.23, *p* < 0.001 for ≤1 h/day; β = 0.18, *p* < 0.01 for ≤2 h/day; β = 0.45, *p* < 0.001 for ≤3 h/day; β = 1.43, *p* < 0.001 for >3 h/day) were positively associated with poor sleep quality, compared with people without evening screen time. Similar results were also observed in the 35-to-49-year age group (β = 0.43, *p* < 0.001 for ≤1 h/day; β = 0.43, *p* < 0.001 for ≤2 h/day; β = 0.62, *p* < 0.001 for ≤3 h/day; β = 1.55, *p* < 0.001 for >3 h/day) and 50-to-64-year age group (β = 0.36, *p* < 0.001 for ≤1 h/day; β = 0.31, *p* < 0.01 for ≤2 h/day; β = 0.61, *p* < 0.001 for ≤3 h/day; β = 1.88, *p* < 0.001 for >3 h/day). However, when analyzing the 18-to-34-year age group, only individuals with ≤1 h/day (β = 0.34, *p* < 0.05) or >3 h/day (β = 1.05, *p* < 0.001) of AEST were at a higher risk of poor sleep quality. In the 65-year-and-older age group, AEST with ≤3 h/day (β = 0.82, *p* < 0.001) and >3 h/day (β = 1.84, *p* < 0.001) were at a higher risk of poor sleep quality.

**TABLE 2:** Multiple linear regression for the factors associated with poor sleep quality in the whole sample (β and its 95% CI).

Variables	Total	18–34 years	35–49 years	50–64 years	≥65 years
Observations	21376 (100.0)	4424 (20.7)	4970 (23.3)	7282 (34.1)	4700 (22.0)
Age (Continuous)	0.06 (0.06, 0.07)***	0.03 (0.01, 0.05)**	0.07 (0.05, 0.09)***	0.06 (0.04, 0.08)***	0.001 (−0.02, 0.02)
Male	−1.03 (−1.15, −0.92)***	−0.47 (−0.65, −0.29)***	−0.57 (−0.78, −0.35)***	−1.36 (−1.58, −1.14)***	−1.42 (−1.70, −1.14)***
Hans (Ref. = Others)	−0.23 (−0.42, −0.04)**	−0.49 (−0.79, −0.20)***	−0.13 (−0.48, 0.23)	−0.33 (−0.68, 0.03)	−0.08 (−0.54, 0.38)
**Education (Ref. = High school/above)**					
Illiteracy	0.66 (0.48, 0.84)***	1.30 (0.64, 1.96)***	0.68 (0.20, 1.16)**	0.55 (0.24, 0.85)***	0.27 (−0.19, 0.72)
Elementary	0.37 (0.22, 0.51)***	0.16 (−0.13, 0.44)	0.62 (0.34, 0.89)***	0.27 (0.02, 0.52)*	0.09 (−0.34, 0.51)
Middle school	0.16 (0.04, 0.29)**	0.10 (−0.07, 0.28)	0.30 (0.06, 0.53)*	0.22 (−0.00., 0.45)	−0.03 (−0.46, 0.41)
**Married Status (Ref. = Others)**					
Unmarried	−0.38 (−0.66, −0.11)***	−1.29 (−1.99, −0.60)***	−0.43 (−1.24, 0.38)	−0.80 (−1.54, −0.07)*	−0.19 (−1.22, 0.85)
Married	−0.36 (−0.55, −0.16)***	−1.04 (−1.71, −0.37)**	−0.96 (−1.60, −0.33)**	−1.05 (−1.47, −0.64)***	−0.27 (−0.57, 0.03)
Rural region	0.02 (−0.09, 0.14)	−0.48 (−0.68, −0.29)***	−0.19 (−0.41, 0.04)	0.20 (−0.001, 0.40)	0.49 (0.20, 0.77)***
Smoking	0.24 (0.10, 0.37)**	0.30 (0.08, 0.52)**	0.24 (0.000, 0.48)*	−0.003 (−0.25, 0.24)	0.24 (−0.11, 0.59)
Drinking	−0.01 (−0.14, 0.12)	0.19 (−0.03, 0.40)	0.09 (−0.14, 0.31)	−0.04 (−0.28, 0.20)	−0.54 (−0.89, −0.20)**
Physical activity					
(≥ 3 times/week)	−0.41 (−0.50, −0.32)***	−0.45 (−0.60, −0.31)***	−0.41 (−0.58, −0.25)***	−0.57 (−0.74, −0.39)***	−0.22 (−0.45, 0.02)
**AEST (Ref. = 0)**					
≤1 h/day	0.23 (0.11, 0.34)***	0.34 (0.06, 0.61)*	0.43 (0.19, 0.68)***	0.36 (0.15, 0.57)***	−0.15 (−0.43, 0.13)
≤2 h/day	0.18 (0.05, 0.31)**	0.26 (−0.003, 0.52)	0.43 (0.19, 0.68)***	0.31 (0.09, 0.54)**	−0.12 (−0.42, 0.17)
≤3 h/day	0.45 (0.28, 0.62)***	0.25 (−0.04, 0.54)	0.62 (0.29, 0.94)***	0.61 (0.26, 0.96)***	0.82 (0.35, 1.30)***
>3 h/day	1.43 (1.15, 1.71)***	1.05 (0.67, 1.43)***	1.55 (0.94, 2.16)***	1.88 (1.30, 2.45)***	1.84 (1.05, 2.62)***
Constant	2.23 (1.86, 2.61)***	4.05 (3.07, 5.03)***	1.68 (0.59, 2.76)**	3.42 (2.14, 4.70)***	6.33 (4.68, 7.98)***
R^2^	0.12	0.05	0.04	0.06	0.06

AEST denotes average evening screen time. CI denotes confidential interval. *p < 0.05; **p < 0.01; ***p < 0.001.

As the line charts showed the reverse S-shaped and J-shaped associations between AEST and poor sleep quality, multiple linear regression analyses were further conducted to explore the associations among participants with evening screen time. The results showed that screen time only >3 h/day was statistically significant in the 18-to-34-year age group (β = 0.72, *p* < 0.001), 35-to-49-year age group (β = 1.11, *p* < 0.001), and 50-to-64-year age group (β = 1.52, *p* < 0.001), compared with participants using screen ≤1 h/day in the evening. In the 65-year-and-older age group, ≤3 h/day (β = 0.92, *p* < 0.001) and >3 h/day (β = 1.90, *p* < 0.001) were at a higher risk of poor sleep quality, compared with participants using screen ≤1 h/day in the evening. The detailed information is summarized in Table 3.

**TABLE 3:** Multiple linear regression for the factors associated with poor sleep quality among participants with evening screen using (β and its 95% CI).

Variables	Total	18–34 years	35–49 years	50–64 years	≥65 years
Age (Continuous)	0.06 (0.06, 0.06)***	0.03 (0.01, 0.05)*	0.07 (0.05, 0.09)***	0.07 (0.05, 0.09)***	0.01 (−0.01, 0.03)
Male	−1.04 (−1.16, −0.93)***	−0.48 (−0.67, −0.28)***	−0.55 (−0.78, −0.31)***	−1.28 (−1.53, −1.03)***	−1.37 (−1.70, −1.04)***
Hans (Ref. = Others)	−0.24 (−0.42, −0.05)*	−0.49 (−0.80, −0.18)**	−0.14 (−0.52, 0.23)	−0.20 (−0.59, 0.19)	−0.34 (−0.86, 0.19)
**Education (Ref. = High school/above)**					
Illiteracy	0.64 (0.46, 0.83)***	1.25 (0.47, 2.03)**	0.50 (−0.06, 1.06)	0.70 (0.35, 1.05)***	−0.07 (−0.59, 0.44)
Elementary	0.36 (0.22, 0.51)***	0.19 (−0.12, 0.50)	0.54 (0.25, 0.84)***	0.39 (0.11, 0.67)**	0.01 (−0.47, 0.48)
Middle school	0.16 (0.04, 0.29)**	0.11 (−0.07, 0.29)	0.32 (0.07, 0.57)*	0.30 (0.05, 0.55)*	−0.17 (−0.64, 0.31)
**Married Status (Ref. = Others)**					
Unmarried	−0.38 (−0.66, −0.11)**	−1.15 (−1.89, −0.41)**	−0.52 (−1.41, 0.37)	−0.94 (−1.82, −0.06)*	−0.53 (−1.79, 0.72)
Married	−0.35 (−0.54, −0.15)***	−0.86 (−1.57, −0.15)*	−0.96 (−1.64, −0.27)**	−1.01 (−1.51, −0.51)***	−0.47 (−0.83, −0.11)*
Rural region	0.01 (−0.10, 0.12)	−0.46 (−0.66, −0.26)***	−0.21 (−0.45, 0.02)	0.17 (−0.05, 0.39)	0.50 (0.18, 0.82)**
Smoking	0.24 (0.11, 0.38)***	0.35 (0.12, 0.59)**	0.25 (−0.01, 0.50)	−0.02 (−0.30, 0.25)	0.33 (−0.07, 0.71)
Drinking	−0.002 (−0.13, 0.13)	0.16 (−0.07, 0.38)	0.02 (−0.23, 0.26)	−0.05 (−0.32, 0.22)	−0.47 (−0.86, −0.08)*
Physical activity					
(≥3 times/week)	−0.41 (−0.50, −0.32)***	−0.46 (−0.61, −0.31)***	−0.34 (−0.52, −0.17)***	−0.36 (−0.56, −0.17)***	−0.08 (−0.36, 0.21)
**AEST (Ref. = ≤ 1 h/day)**					
≤2 h/day	0.03 (−0.07, 0.13)	−0.08 (−0.26, 0.11)	−0.01 (−0.20, 0.18)	−0.05 (−0.25, 0.15)	−0.002 (−0.28, 0.28)
≤3 h/day	0.29 (0.14, 0.45)***	−0.08 (−0.30, 0.14)	0.18 (−0.10, 0.46)	0.24 (−0.08, 0.57)	0.92 (0.47, 1.37)***
>3 h/day	1.29 (1.02, 1.56)***	0.72 (0.39, 1.06)***	1.11 (0.53, 1.69)***	1.52 (0.97, 2.08)***	1.90 (1.15, 2.64)***
Constant	2.42 (2.06, 2.78)***	4.24 (3.22, 5.27)***	2.19 (1.06, 3.33)***	2.75 (1.30, 4.21)***	6.05 (4.11, 7.99)***
R^2^	0.12	0.05	0.03	0.06	0.06

AEST denotes average evening screen time. CI denotes confidential interval. *p < 0.05; **p < 0.01; ***p < 0.001.

## Discussion

In this population-based, cross-sectional study, the results supported that sleep quality decreased with the age, and AEST was also different among different age groups. In general, the association between sleep quality and AEST was supported in our findings. However, the different recommended screen time readings were also found in different age groups. In the 18-to-34-year age group, more than 3 h/day or less than 1 h/day of AEST was significantly associated with poor sleep quality. In the 35-to-49-year and 50-to-64-year age groups, AEST was positively associated with poor sleep quality, and those with more than 3 h/day of AEST were at a higher risk of poor sleep quality. In the 65-year-and-older age group, only more than 2 h/day of AEST was associated with poor sleep quality.

The first observation in this study was about the current situation and associations between sleep quality and AEST among different age groups, and there were no surprising findings for these results. Regarding sleep quality, our results supported that sleep quality decreased with age, which has been identified in many previous studies (35–37). The results also supported that a higher percentage of young people used screen-based products in the evening, which was also consistent with other findings (38, 39). In the whole sample with a wide range of age groups, AEST was also positively associated with poor sleep quality, which was also supported in previous findings (20, 21).

In this study, the associations between AEST and sleep quality were analyzed in different age groups. In the 18-to-34-year age group, less than 1 h/day or more than 3 h/day of AEST was significantly associated with poor sleep quality, and a reverse S-shaped relationship between AEST and poor sleep quality was shown for this age group. In the younger age group, they are characterized by a higher risk of addictive symptoms caused by screen time (40). Sleep complaints, as one of the prodromal symptoms of addictive symptoms, are observed because of more AEST (41). In contrast, the associations between sleep quality and 1–3 h of AEST were not supported in our results. It may be explained by the fact that a moderate screen time may improve happiness or mood relaxation (42, 43), and both happiness and mood relaxation can improve sleep quality. However, when AEST increased to more than 3 h, the risk of poor sleep quality was significantly increased. This is caused by the fact that the long-term screen time results in delayed bedtime and wake-up time, which has been identified among adolescents and college students (44–46).

In the 35-to-49-year and 50-to-64-year age groups, similar results about the association between AEST and sleep quality were supported in this study. In these two age groups, participants with any evening screen time were at a higher risk of poor sleep quality, compared with people without AEST, and the risk further increased for people with more than 3 h of AEST. This may be caused by the fact that people in these age groups have higher work stress (47), and they need more time to relax. Although evening screen time can be seen as a kind of relaxation, they may be too tired to have evening screen time. This may cause a persistently positive association between AEST and poor sleep quality.

In the 65-year-and-older age group, our results supported that only more than 2 h/day of AEST was associated with poor sleep quality, and the results also supported a J-shaped relationship between AEST and sleep quality. Actually, a study also reported the J-shaped relationship between screen time and sleep quality (22). In China, people aged 65 years and above have retired, and they need some recreational activities in their lives. Evening screen time, a kind of recreational method may be helpful for them to relax their mood and further improve their sleep quality. However, when evening screen time is at a higher level, it may be a risk factor for sleep quality. The reasons are similar to the other age groups.

The main aim of this study was to investigate the association between AEST and sleep quality among different age groups. Although the higher level of AEST (>3 h/day) was associated with poor sleep quality in all the analyzed age groups, there were some differences among different age groups. As we introduced, one of the explanations is the age differences between the evening screen time and sleep quality. The other reason may be the different work stress and age characteristics. As we have discussed earlier, we do not explain it here again.

Some limitations should be considered when we interpret the findings of this study. First, any causal relationships cannot be inferred for the association between AEST and sleep quality because of the cross-sectional design. Second, AEST and sleep quality were evaluated by the participants’ self-report, and some bias caused by the evaluation methods cannot be avoided. Third, there are a majority of factors associated with AEST and sleep quality, and only confounders about sociodemographic variables and health-related behaviors were analyzed in this study, which may also cause some bias in the findings. Considering these limitations, a cohort or experimental research with a more accurate evaluation of AEST and sleep quality will be helpful for us to explore the causal relationship between them in the future.

Despite these limitations, to the best of our knowledge, this is the first population-based study, which distinguished the associations between AEST and sleep quality among different age groups. The results also remind us that the associations between AEST and sleep quality among different age groups are different. In the 18-to-34-year age group, controlling AEST is necessary to improve their sleep quality. In the 35-to-49-year and 50-to-64-year age groups, AEST was positively associated with poor sleep quality, which should be paid attention. In the 65-year-and-older age group, some moderate time of AEST is not associated with sleep quality, but a higher level of AEST should be controlled to improve their sleep quality.

## Data Availability Statement

The raw data supporting the conclusions of this article will be made available by the authors, without undue reservation.

## Ethics Statement

The studies involving human participants were reviewed and approved by the Institutional Review Board (IRB) of Hebei Mental Health Center. The patients/participants provided their written informed consent to participate in this study.

## Author Contributions

LS analyzed the data and wrote the draft. KL and LZ commented on the manuscript. YZ designed the study and commented on the draft of this manuscript. All authors read and approved the final manuscript.

## Conflict of Interest

The authors declare that the research was conducted in the absence of any commercial or financial relationships that could be construed as a potential conflict of interest.

## Publisher’s Note

All claims expressed in this article are solely those of the authors and do not necessarily represent those of their affiliated organizations, or those of the publisher, the editors and the reviewers. Any product that may be evaluated in this article, or claim that may be made by its manufacturer, is not guaranteed or endorsed by the publisher.
